# Molding acoustic, electromagnetic and water waves with a single cloak

**DOI:** 10.1038/srep10678

**Published:** 2015-06-09

**Authors:** Jun Xu, Xu Jiang, Nicholas Fang, Elodie Georget, Redha Abdeddaim, Jean-Michel Geffrin, Mohamed Farhat, Pierre Sabouroux, Stefan Enoch, Sébastien Guenneau

**Affiliations:** 1 Department of Mechanical Engineering, Massachusetts Institute of Technology, 77 Massachusetts Avenue, Cambridge, MA 02139-4307; 2 Aix-Marseille Université, CNRS, Centrale Marseille- Institut Fresnel, Campus universitaire de Saint-Jérôme, 13013 Marseille, France; 3 Division of Computer, Electrical, and Mathematical Sciences and Engineering, King Abdullah University of Science and Technology (KAUST), Thuwal 23955-6900, Saudi Arabia

## Abstract

We describe two experiments demonstrating that a cylindrical cloak formerly introduced for linear surface liquid waves works equally well for sound and electromagnetic waves. This structured cloak behaves like an acoustic cloak with an effective anisotropic density and an electromagnetic cloak with an effective anisotropic permittivity, respectively. Measured forward scattering for pressure and magnetic fields are in good agreement and provide first evidence of broadband cloaking. Microwave experiments and 3D electromagnetic wave simulations further confirm reduced forward and backscattering when a rectangular metallic obstacle is surrounded by the structured cloak for cloaking frequencies between 2.6 and 7.0 GHz. This suggests, as supported by 2D finite element simulations, sound waves are cloaked between 3 and 8 KHz and linear surface liquid waves between 5 and 16 Hz. Moreover, microwave experiments show the field is reduced by 10 to 30 dB inside the invisibility region, which suggests the multi-wave cloak could be used as a protection against water, sonic or microwaves.

Eight years ago, two seminal papers by Pendry, Schurig and Smith^1^ and Leonhardt^2^ have revolutionized the way we think about control of electromagnetic waves. These authors proposed to mold the flow of light through space coordinate transformations. As an illustration of this transformational optics (TO) tool, a design of a spherical invisibility cloak was proposed in^1^ by mapping a sphere onto a hollow sphere. The shell surrounding the hole consists of an anisotropic heterogeneous metamaterial described by spatially varying, matrix valued, permittivity and permeability deduced from a formula based on the invariance of Maxwell’s equations under geometric transforms. Such a route to cloaking can be viewed as analogous to what Einstein’s equations teach us in relativity^3^ light trajectories follow geodesics associated with the space (-time) metric. Interestingly, in^2^ conformal mappings were used, which preserve right angles of the metric and thus avoid the anisotropic nature of coefficients in the transformed equations, but this conformal optics (CO) approach to cloaking is constrained by wavelengths small compared to the object to hide. Both TO and CO approaches have fueled metamaterials’ research, with notably the experimental proof of concept of a cylindrical cloak consisting of concentric layers of split ring resonators of varying size molding the trajectory of transverse magnetic waves around a copper cylinder at 8.5 GHz via artificial anisotropic permeability^4^. The constraint on extreme material parameters has been since then relaxed with the advent of carpet cloaks^5^ which is somewhat a mitigation of TO and CO. Their design is based upon quasi-conformal maps, which considerably reduce anisotropy of the transformed medium and have thus made possible fabrication and characterization of broadband three-dimensional ground cloaks at optical wavelengths^6^,^7^ and even a nearly perfect two-dimensional omnidirectional cloak in the microwave regime^8^.

In the tracks of electromagnetic cloaks, control of acoustic and water wave trajectories has been proposed^9^,^10^,^11^,^12^,^13^ with acoustic metamaterials. It is also possible to detour elastodynamic waves^14^,^15^,^16^, although this requires a fairly exotic (possibly asymmetric) elasticity tensor for fully coupled pressure and shear waves. This is due to the fact that when one moves into the area of elastodynamic waves, governing equations become much less tractable as pressure and shear waves are inherently coupled in structured solids. Another difficulty intrinsic to the tensor nature of the Navier equations is that they do not behave nicely upon a change of coordinates^14^.

In contrast to Maxwell’s equations, the form of Navier equations is not preserved under geometric transforms such as space folding, whereby the image plane of a flat lens with a negative refractive index is expected to map onto its source plane^3^, and this explains perhaps why Pendry’s perfect lens^17^ can only be extended to specific elastodynamic wave polarizations, such as subwavelength focusing of Lamb waves in thin plates^18^. Let us note in passing that some form of external cloaking can be achieved via space folding for a finite set of dipoles located in the close neighborhood of a perfect lens^19^.

Notwithstanding the fact that cloaking displays some universal features such as enhanced control of wave wavefronts (one might say a wave is a wave), some word of caution should be made regarding invisibility versus protection in various physical contexts. Indeed, cloaking for diffusion of heat^20^,^21^,^22^ shows one can make a hole in the transformed space and achieve invisibility (i.e. control of isothermal curves in the case of heat flux management). However, waves and heat behave in different ways in the central region of invisibility cloaks: the amplitude of waves should vanish inside the core of acoustic and electromagnetic cloaks, while the temperature might still undergo some elevation inside the core of thermal cloaks. In the present report, we would like to look at the field inside an electromagnetic cloak. We recall in Fig. 1(a–c) the experimental setup and earlier proof of cloaking via reduction of backscattering for the waterwave cloak introduced in^10^, see photo in Fig. 1(e), for an acoustic source placed in its close neighborhood at 10 Hz. We would like to investigate the electromagnetic response of this cloak when it is placed in an anechoic chamber for microwaves, as schematically shown in Fig. 1(d), as well as for pressure waves, see Fig. 1(f). In order to assess the cloaking efficiency of the cloak in the microwave frequency range [2.6,6.0] GHz, we numerically computed the total radar cross section (RCS) of the cloak, a metal obstacle, and the cloak surrounding the metal obstacle using the CST Microwave Studio software, see Fig. 2. We further computed the counterpart of the total RCS of the cloak for pressure waves using the COMSOL Multiphysics software that indicate cloaking within the frequency range [1,5.8] KHz, see Fig. 3. In the microwave experiment, a ridged horn antenna placed a distance of 24 cm behind the cloak generates transverse electromagnetic waves between 0.8 and 18 GHz which are measured with a magnetic probe (mostly) sensitive to vertical magnetic fields. This allows us to measure one component of the magnetic field in the plane 5 mm above the cloak, outside and inside the cloak, both in magnitude and phase, see Figs. 4&5. One of our findings is that the field inside the core of the cloak is considerably reduced at the cloaking frequencies, as it transpires in the magnetic field’s intensity profiles in Figs. 6&7, and the 2D plots of magnetic field’s phase and intensity in Figs. 5,8&9. This suggests some form of protection for the object placed inside the cloak (a square metallic bar in the microwave experiment, see Fig. 1, and a glass bottle in the acoustic experiment). We also show in this paper that the cloak works equally well for pressure waves in air, see Figs. 3&4. This is due to the fact that the same mathematical model (Helmholtz equation with Neumann boundary conditions set at the structural elements of the aluminium cloak) is valid for transverse electric waves (structural elements behave like infinite conducting inclusions in microwaves), pressure waves (they behave like rigid inclusions in air) and waterwaves (no flow conditions). We note that the microwave cloak of Schurig *et al*.^4^ works for Transverse Magnetic (Ez) polarization, but that of Kanté & de Lustrac works for Transverse Electric (Hz) polarization^23^, simply by changing the orientation of split ring resonators. However, resonances of SRRs make these cloaks narrowband. We stress that our cloak works via homogenization^24^,^25^,^26^,^27^,^28^,^29^,^30^ and cloaking is here simply a consequence of a specific geometry (perforated concentric domain) as we shall see in the sequel. If one were to design an acoustic cloak based on acoustic SRRs, a good way forward would be to implement the design in^31^. Note that our approach to cloaking is also notably different from that in^32^, which is based on a reverse engineering algorithm allowing for considerable reduction of acoustic scattering at a given frequency, but our work is somewhat reminiscent to the approach of^33^ for the design of ground plane acoustic cloaks via effective anisotropy. On a larger scale, our work might also inspire designs of seismic cloaks^34^.

## Results

### Theory and numerics

We proceed with the mathematical approach of homogenization^29^,^30^, which amounts to replacing a structured material by an effective medium that captures the essential wave phenomena for wave wavelengths large compared to the typical heterogeneity size.

It can be shown, that sound waves with a wavelength much larger (in practice three times larger) than the largest structural elements of the cloak are governed by the following homogenized wave equation (written in polar coordinates)


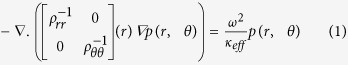


where the effective tensor of density is given by


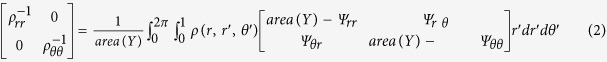


Here, 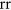 are periodic potentials given by


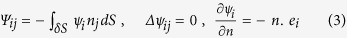


where n is the outer unit normal to the boundary *δS* of the inclusion S in a unit cell Y.

The inverse of the effective bulk modulus is such that


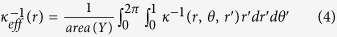


Here r’ and θ’ are radial and azimuthal microscopic variables, and it should be noted that 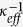 varies with the macroscopic variable r.

In a similar way, transverse electric waves are solution of the following homogenized Maxwell’ equation in the cloak (see also supplemental material):


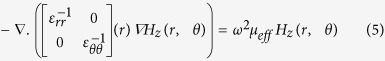


where 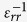 and 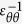 are the coefficients of the effective tensor of permittivity which are also given by (2) (replacing ρ by μ in the equation) and *μ*_*eff*_ is the effective permeability which is such that 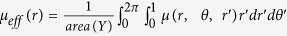. Note here that (4) is simply derived from the vector Maxwell equation by considering a magnetic field *H *= (0,0,*H*_*z*_).

It transpires from the integral expression of *μ*_*eff*_ above that *μ*_*eff*_ < *μ*_*0*_ i.e. that the effective permeability has slightly lower, yet strictly positive, values than permeability of vacuum and its spatial radial variation is small. Hence, one can safely divide by *μ*_*eff*_ throughout in (5). In the same way, from the expression in (4) one can multiply throughout by *κ*_*eff*_ in (1). This leads to reduced effective parameters.

It can be shown that the reduced effective parameters in (1)-(4) are good approximations for well-known transformed density and permittivity in their reduced forms i.e.


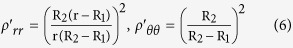



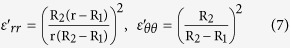


In which case the transformed bulk modulus and permeability can be normalized to 1.

**Figure 1:**
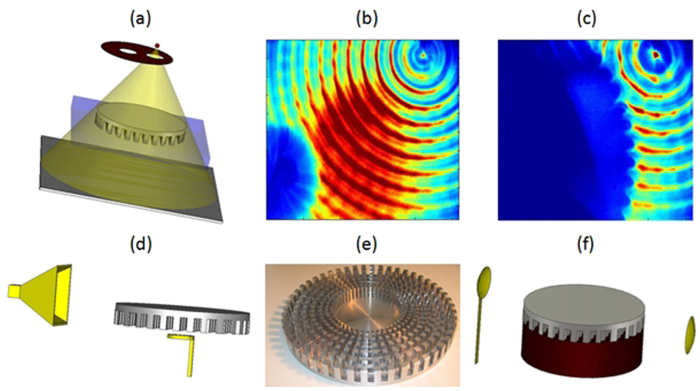
Overview of earlier results on waterwave cloak and new acoustic and electromagnetic experimental setups. (**a**) artistic view of the experimental setup for linear surface water waves showing a source of light modulated by a perforated rotating disc, which illuminates a transparent vessel containing the liquid and the cloak, providing snapshots of wave pattern on a screen; (**b**-**c**) experimental results of reduced backscattering adapted from^10^ for a concentric liquid surface wave of frequency 10 Hz interacting with a rigid cylinder (7.6 cm in diameter) on its own (**b**) and the structured waterwave cloak (20 cm in diameter) (**c**); (**d**) artistic view of the experimental setup of the waterwave cloak tested in Fresnel Institute’s anechoic chamber, with a rigded horn antenna generating transverse electric microwaves with frequencies ranging from 1 to 6 GHz; the longitudinal magnetic field is measured with a magnetic probe (underneath the cloak); (**e**) photo of the cloak; (**f**) artistic view of the experimental setup in MIT for the waterwave cloak tested for pressure acoustic waves generated by a speaker on the left, which are measured with a microphone mounted on a horizontal translational stage on the right.

**Figure 2:**
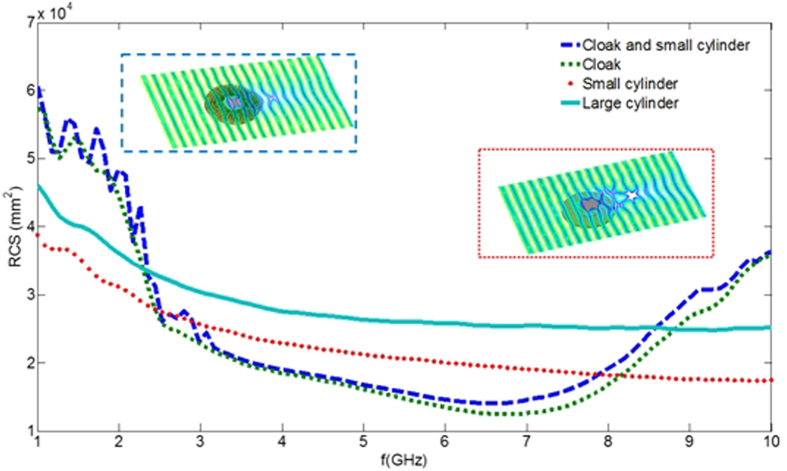
Numerical computation of radar cross section (RCS) for cloak and cylindrical metal obstacles versus frequency (three-dimensional electromagnetic simulations with Computer Simulation Technology Microwave Studio). A small metallic obstacle surrounded by cloak (dashed dark blue curve) is nearly superimposed with RCS of cloak on its own (dotted green curve), and lower than RCS of small metallic obstacle (dotted red curve), which is itself smaller than RCS of large metallic obstacle of same diameter as the cloak (solid light blue curve) from 3 GHz to 7.8 GHz. Two insets further show isovalues of the real part of the longitudinal magnetic field for a plane wave incident at 3.5 GHz from the left on the small obstacle surrounded by the cloak –see inset marked with dashed blue lines—and on the small obstacle on its own –see inset with red dotted lines—, the latter displaying stronger forward scattering.

Modeling of the invisibility cloak has been carried out using the commercial finite elements package COMSOL MULTIPHYSICS. In practice, the cloak consists of a ring of inner radius R_1_ = 4.1 cm and outer radius R_2_ = 10 cm which has been first divided into 14 layers whose thickness varies with respect to r as 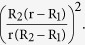 One layer in two has been further divided in 100 identical angular sectors along the azimuthal direction θ as shown in Fig. 1(c). This alternation of homogeneous and structured layers has been optimized by solving the annex problem (3) with COMSOL, see^10^ for the numerical representation of periodic potential *Ψ*_*ij*_ solution of this annex problem in the context of linear surface liquid waves. This leads to the reduced effective parameters in (6) and (7). In order to quantitatively assess the cloaking efficiency of the metamaterial, following the procedure proposed in^35^, we numerically compute its total radar cross section (RCS)^36^,^37^ with the software CST Microwave Studio. We show in Fig. 2 the electromagnetic RCS of the cloak on its own, of the cloak when its surrounds a small metal cylinder (see inset marked by dashed blue line in Fig. 2), of the small metal obstacle (see inset marked by dotted red line in Fig. 2), and of a large metal obstacle with same radius R_2_ as the cloak. Moreover, in all these configurations, we consider the ground plane (5 mm thick) which is part of the cloak: this ground plane cannot be omitted in the three dimensional electromagnetic experimental setup. The RCS of the cloak (dark blue dashed curve) and of the small obstacle surrounded by the cloak are nearly superimposed in the frequency interval [2.6,7.8] GHz and much lower than the small obstacle on its own (dotted red curve). We have numerically checked that the shape and size of the small obstacle matters very little (provided the obstacle can fit within the central region of inner radius R_1_ of the cloak).

**Figure 3:**
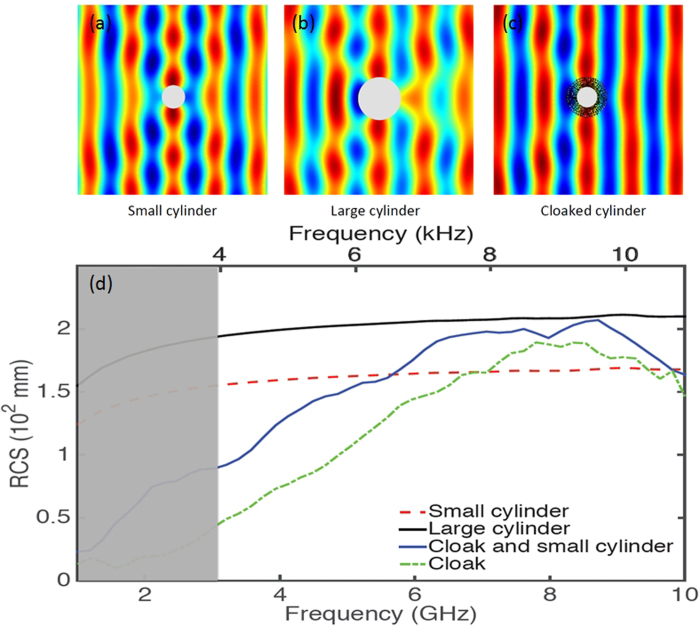
Numerical computation of radar cross section (RCS) for cloak and cylindrical rigid obstacles versus frequency (two-dimensional simulations with Comsol Multiphysics). A small infinite conducting (resp. rigid for pressure waves) obstacle surrounded by cloak (solid dark blue curve) is slightly above RCS of cloak on its own (dotted green curve), but lower than RCS of small infinite conducting (resp. rigid) obstacle (dotted red curve), which is itself smaller than RCS of large infinite conducting (resp. rigid) obstacle of same diameter as the cloak (solid black curve) up to 7 GHz (resp. 8 KHz for pressure waves). Panels (**a**), (**b**), (**c**) further show the real part of the transverse magnetic (resp. pressure) field for a plane wave incident at 4.5 GHz (resp. 5 KHz) from the left on the small obstacle surrounded by the cloak on the small obstacle on its own (**a**), on the large obstacle (**b**), and on the cloaked obstacle (**c**).

We further show in Fig. 3 the electromagnetic and acoustic RCS computed with COMSOL MULTIPHYSICS using the 2D Helmholtz equation, which demonstrates cloaking for microwaves up to 7 GHz and sound waves up to 8 KHz (the lower frequency bound is irrelevant as the wave wavelength becomes larger than the cloak’s height): These two-dimensional finite element simulations are valid for both sonic and transverse electric microwaves provided we make the assumption that the cloak is invariant along the vertical direction (see also Supplemental Material for RCS of linear surface water waves, which further confirms earlier results of^10^). We simply solve the Helmholtz equation with Neumann boundary conditions on the cloak’s structural elements, which is a good model for rigid (resp. infinite conducting) inclusions in the context of pressure (resp. transverse electric) waves as announced in the introduction.

**Figure 4:**
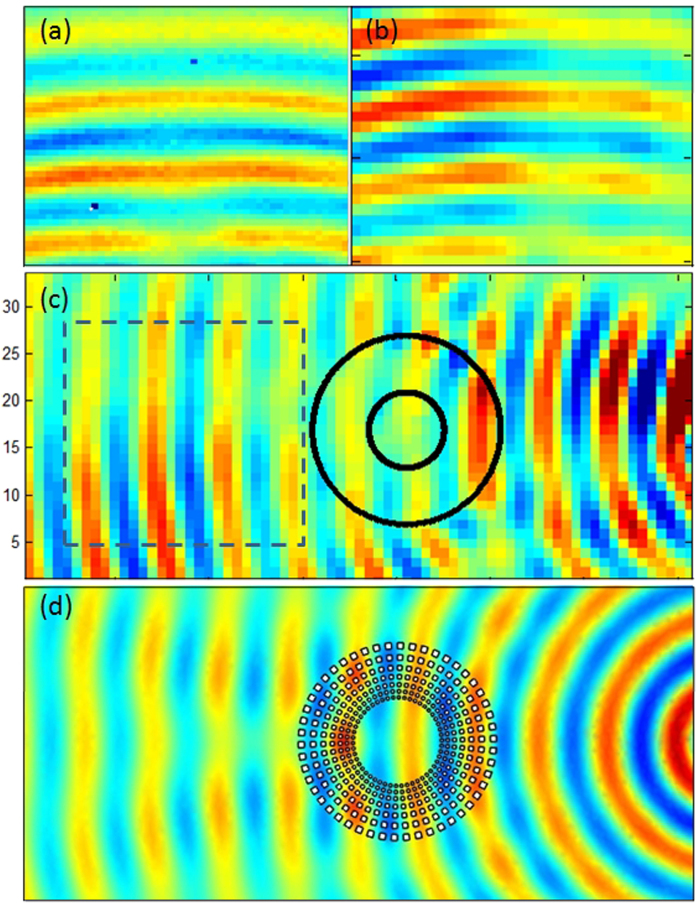
Acoustic and electromagnetic wave experiments versus finite element simulation. (a,b) Experimental results in forward scattering for pressure waves (**a**) and transverse electric microwaves (**b**) for the same cloak containing a large glass bottle obstacle (**a**) and a small square metallic obstacle (**b**); The source is located 24 cm from the center of the cloak and its frequency lies inside the cloaking zone (5 KHz for pressure waves in (**a**) and 4.350 GHz for microwaves in (**b**); The real part of the pressure field in (**a**) and of the longitudinal magnetic field in (**b**) is measured within the inset of panel (**c**)-- see area marked with dashed lines--, which shows experimental results in backward and forward scattering for microwaves; (**d**) Comparison with finite element simulation, where Neumann (rigid for pressure field and infinite conducting for longitudinal magnetic field) boundary conditions are set on each inclusion. Cloaking is achieved as it transpires from the nearly unperturbed phase in both scenarios.

For the same 2D setup as in Fig. 3, we then show in Fig. 4 the total field scattered by a time harmonic acoustic source (resp. electromagnetic source) placed a few wavelengths away from the cloak at frequency 5 KHz (resp. 4.5 GHz), see Fig. 4 upper left panel (resp. upper right panel). The wave wavefronts are neatly reconstructed behind the cloak, as one can observe in the insets marked with the dashed parameter in both panels. The inset marked with the dashed perimeter in the middle panel is used for experimental comparisons between the pressure and microwave experiments in forward scattering in the upper panels, but both forward and backward scattering are shown for microwaves in the middle panel, with a good agreement with numerical simulation in the lower panel.

### Experiments

Acoustic experiments were performed in MIT (Boston, USA) for sound waves and in Fresnel Institute (Marseille, France) for microwaves. We considered the two-dimensional Helmholtz model for the comparison between theory and acoustic experiments, therefore it came as a good surprise that our intuition was right: experiments in Fig. 4 clearly demonstrate that although the cloak’s height is not large compared to the wave wavelength, it behaves as expected. An object (a large glass bottle in MIT and a small square metallic obstacle in Fresnel Institute) is rendered invisible by the structured aluminium cloak. In Fig. 4(a–b), we emphasize that slights discrepancies between phase of pressure and longitudinal magnetic field can be attributed to scattering induced by the free space experimental setups.

The experimental electromagnetic setup at Institut Fresnel is actually more in the spirit of that of Kanté *et al*.^23^ which is a nonmagnetic microwave cloak also characterized in free space, than the one of Schurig *et al*.^4^. Note however that both cloaks in^4^,^23^ heavily rely upon resonances of split ring resonators, whereas in our case cloaking is associated with some artificial, geometrical induced, anisotropy. The phase of the pressure field in the left panel of Fig. 3(a) displays strong similarities with that of^11^, although in this paper cloaking was achieved at ultrasonic frequencies in a shallow water experiment (the layer of water helped to guide the pressure waves, unlike for the current experiment). This suggests that although the current sound cloaking experiment was carried out in free space for this report, the cloak effectively controlled the wave path of sonic waves propagating in a layer of air passing through it.

Regarding the electromagnetic cloaking experiment, we were able to monitor both the phase and intensity of the longitudinal component of the magnetic field, in backward and forward scattering, as well as within the cloak. This experimental result clearly shows that the electromagnetic signal is much reduced inside the core of the cloak, where a metal plate is placed. Similar tests (not reported) have been performed for a cloud of metal nails and here again we observed very little electromagnetic field inside the core of the cloak (a reduction of magnetic field intensity by 13.5 to 38 dB at the cloaking frequency 3.550 GHz). This suggests some form of electromagnetic protection for the square metal plate (or another metal object) can be achieved at cloaking frequencies thanks to the cloak. We also observe that transmission is good for the electromagnetic wave propagating though the structured metal cloak. Hence, we not only achieve a good reconstruction of the wave wavefront (the phase), but also preserve its intensity.

## Discussion

**Figure 5:**
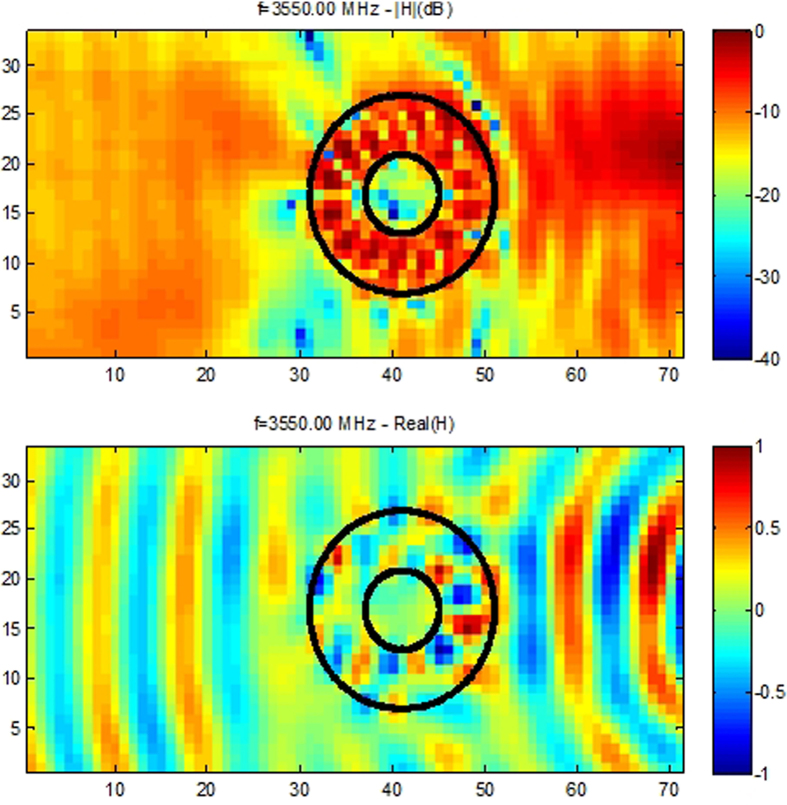
Experimental results in backward and forward scattering for microwaves. The normalized modulus (upper panel) and the normalized real part (middle panel) of the magnetic field is measured within the rectangular domain shown in lower panel. The frequency of the source is 3.550 GHz. One notes that the magnetic field intensity is reduced by 18 dB at the center of the cloak with a reduction varying in the range [13.5,38]dB inside the inner disc. The field is enhanced inside the corona and both wavefront and field amplitude are nicely reconstructed behind the cloak. Notably the measured intensity is −11 dB and −1 dB at the left and right sides of the panel, respectively. See also Figure 5 for the profile of the field along the median passing through the center of the cloak. The square metallic obstacle inside the invisibility region is both invisible and protected from microwaves. Similar results hold in the cloaking interval [2.6,6]GHz, see Figs. 7&8.

One of the main features of the cloak is that it works through field averaging and only one phase (vacuum) need be considered in the homogenization and computational results to achieve a good prediction of experimental results. The underlying mechanism (artificial anisotropy) is so simple that it makes the cloak multiphysics: Two-dimensional numerical simulations with COMSOL MULTIPHYSICS show by a simple normalization of the wave frequency in (1) or (5) that cloaking should be at work between 5 and 16 Hz for surface liquid waves in the condition of^10^, see Fig. S4 of Supplemental Material (the corresponding surface water wave frequency should be 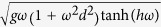 where g denotes the acceleration caused by gravity, d is the liquid capillarity length and h is the liquid depth), between 3 and 8 KHz for sound waves and between 2.6 to 7 GHz for microwaves. We note that the latter interval for cloaking frequencies is slightly shifted compared to that of [2.6,7.8] GHz found via the CST computation of RCS in Fig. 2, what can be attributed to 3D effects (the cloak’s size in the vertical direction is in fact comparable to the electromagnetic wave wavelength) and the presence of a ground plane (supporting the pillars of the cloak). Therefore, our cloak cannot be considered in the strict sense as two-dimensional and our 2D homogenization and numerical models should be considered only as an approximation of full wave models. Experiments shown in Figs. 4 and 5 confirm that the range of cloaking frequencies from 2.6 to 6 GHz (see also Figs. 8 and 9 for other frequencies): Although ridged horn antenna generates transverse electromagnetic waves between 0.8 and 18 GHz, our vector network analyzer only works between 50 KHz and 6 GHz.

**Figure 6:**
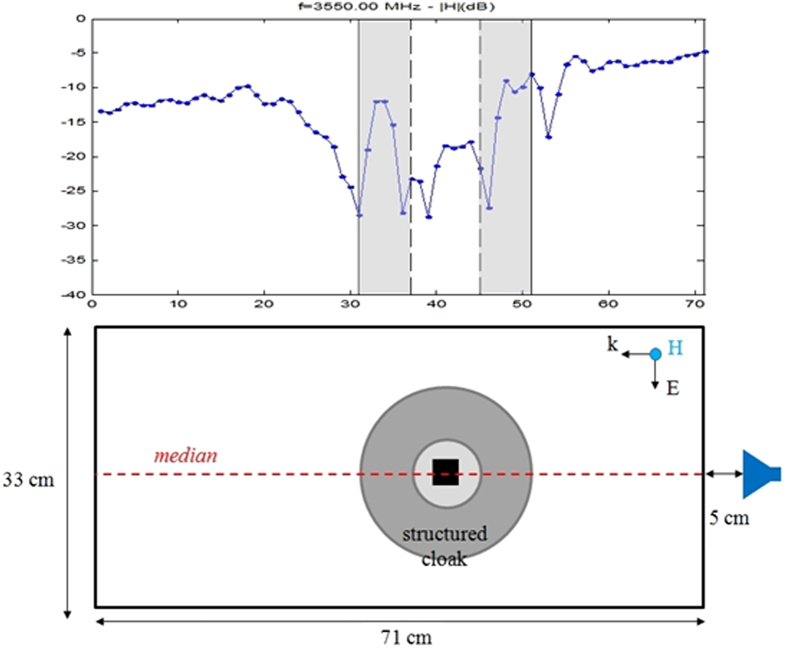
Experimental results in backward and forward scattering for microwaves. The normalized modulus (upper panel)of the magnetic field is measured along the median shown in lower panel. The frequency of the source is 3.550 GHz. One notes that the magnetic field intensity is reduced by 18 dB at the center of the cloak with a reduction up to 30 dB inside the inner disc. The field is enhanced inside the corona and its intensity in front and behind the cloak is close to what it would be in free space. Similar results hold in the cloaking interval [2.6,6]GHz, see Fig. 6.

**Figure 7:**
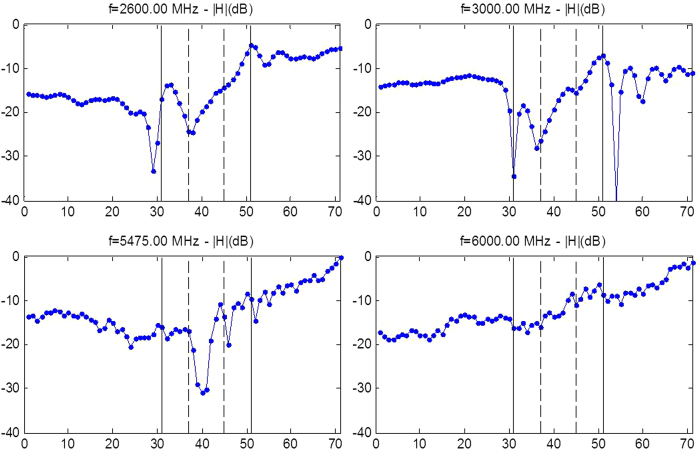
Experimental results for microwaves at four representative frequencies within the cloaking interval [2.6,6]GHz. The normalized modulus (upper panel) of the magnetic field is measured along the median passing through the center of the cloak as in Fig. 4 for the source at 2.6, 3, 5.475 and 6 GHz. One notes that the magnetic field intensity is reduced by up to 30 dB at the center of the cloak (whose inner and outer boundaries are marked by the solid and dashed vertical lines respectively). Protection is clearly more pronounced at 2.6 GHz than 6 GHz. Only invisibility is achieved for the latter frequency.

We stress that we not only show the phase, but also the intensity of the magnetic field in the experiments. One can see that the phase of the signal is reconstructed behind the cloak in Fig. 5, but more importantly the intensity of the signal is preserved, as is also exemplified in Figs. 6 and 7. The field is also measured within the cloak, and as one would hope it does seem to vanish inside the invisibility region. This is important for potential applications in protection from waves, and notably if one would like to scale up the structure in order to make a cloak for ocean waves.

**Figure 8:**
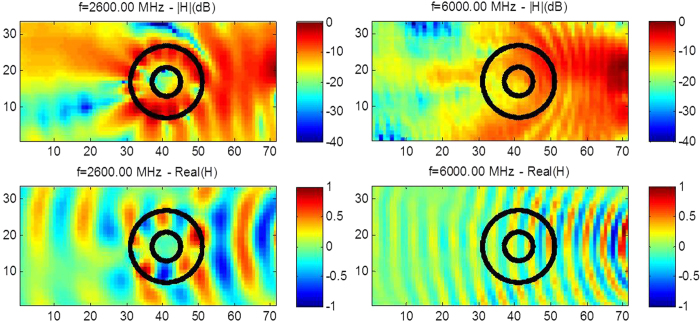
Experimental results for microwaves at the edges of the cloaking interval [2.6,6]GHz. The normalized modulus (upper panel) and the normalized real part (middle panel) of the magnetic field is measured for the source at 2.6 and 6 GHz. One notes that the magnetic field intensity is reduced by 18.5 dB and 13 dB at the center of the cloak with a reduction varying in the range [11.5,35] and dB [8.5,17] dB inside the inner disc, respectively at 2.6 and 6 GHz. The field is enhanced inside the corona and both wavefront and field amplitude is better reconstructed behind the cloak at 6 GHz. Notably the measured intensity is −18 dB and −17 dB at the left side, respectively at 2.6 and 6 GHz and −1 dB at the right side, of the intensity panels. Protection is clearly more pronounced at 2.6 GHz than 6 GHz.

**Figure 9:**
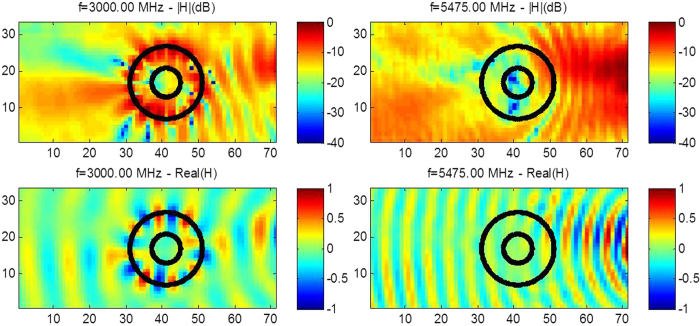
Experimental results for microwaves at two representative frequencies within the cloaking interval [2.6,6]GHz. The normalized modulus (upper panel) and the normalized real part (middle panel) of the magnetic field is measured for the source at 3 and 5.475 GHz. One notes that the magnetic field intensity is reduced by 17 dB and 30 dB at the center of the cloak with a reduction varying in the range [12.5,24]dB and[11,38]dB inside the inner disc, respectively at 3.5 and 5.475 GHz. The field is enhanced inside the corona at 3 GHz and both wavefront and field amplitude is better reconstructed behind the cloak at 5.475 GHz. Notably the measured intensity is −14 dB and −12 dB at the left side, respectively at 3 and 5.475 GHz and −1 dB at the right side, of the intensity panels. Protection is clearly more pronounced at 5.475 GHz than 3 GHz.

The ability of the cloak to control more than one type of waves could have interesting applications in phoxonics. One could also envisage to increase the number of structural elements to improve cloaking even further. Homogenization tells us that the more structural elements, and the smaller their size compared to the wave wavelength, the better approximate cloaking, see Fig. S3&S4 in Supplemental Material for some numerical evidence of this claim. We have thus experimentally demonstrated the first multi-physics cloaking (linear surface water waves, pressure waves and transverse electric waves) with a single cloak. The cloak is broadband, see Figs. 5,8 and 9, relatively easy to fabricate, and offers some form of protection for the object placed inside the invisibility region, see Figs. 6 and 7. We hope our experiments will foster theoretical and experimental efforts in multi-physics cloaking and exploration of invisibility versus protection for different waves. We point out that our cloak is reminiscent of labyrinthine acoustic metamaterials^38^, and it might inspire more elaborate designs of elasto-mechanical^39^ and elasto-electromagnetic^40^ metamaterials. Our cloak could have potential applications in telecommunications (protection against mobile phone radiations) and soundproof devices (humans are most sensitive to sound waves of frequencies between 2 and 5 kHz). The design could also be scaled down in order to achieve cloaking at optical wavelengths, what would require an analysis of cloak’s dispersion, see e.g.^41^. Since the cloak works both in acoustic and microwave domains, it might offer new opportunities to drive and tune one field with the other one: for instance if the fluid within which pressure waves propagate is no longer air but some gas plasma or liquid electrolyte, we might be able to tune the density profile by microwave signals. On larger scales, control of sound and elastic waves could be used in anti-earthquake designs if protection is achieved via cloaking with seismic metamaterials^34^, that is for frequencies below 50 Hz. Finally, we note that preliminary numerical simulations suggest our cloak should also work in the context of management of thermal flux: it can be shown using homogenization methods^42^ that our cloak can be described by an effective conductivity tensor 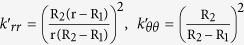 and by a product of effective mass density and specific heat averaged like the inverse of effective bulk modulus in (4). Our cloak could also serve as a prototype thermal metamaterial to mold the flow of heat or even light diffusion in the tracks of^20^,^21^,^22^ and^43^.

## Methods

### Finite element method

COMSOL Multiphysics with “PDE” module has been used to compute the scattered field in Figs. 3&4, with Cartesian perfectly matched layers (PMLs) on either sides of the computational domain to avoid reflections on the computational domain boundaries. Some Neumann boundary conditions are assumed on each structural element of the cloak and the source is set in the form 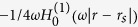 where *r*_*s*_ is the position of the source (with respect to the center of the cloak, origin of the coordinates) and *ω* is the angular wave frequency.

### Homogenization method

The multiple scale technique^30^ allows us to comprehend the cloaking mechanism of the cloak. The main assumption is the decoupling between the microscopic and microscopic scales, respectively associated with fast and slow oscillations of the pressure acoustic field or longitudinal magnetic field. This method leads to a homogenized anisotropic matrix (the effective density, or the effective permittivity^25^, depending upon the type of wave), which is a good approximation of the transformed density, or permittivity, obtained by blowing up a point on a disc^1^. Details on our cloak’s design via numerical solution of the annex problem (3) in the context of linear surface liquid waves can be found in^10^.

### Experimental set-up for microwaves

The microwaves experimental setup was positioned in an anechoic chamber (3 m long, 3 m wide and 3 m high). A ridged horn antenna [1 GHz–18 GHz] was positioned at 24 cm from the structure cloak (Fig. 6). A rectangular metallic obstacle was inside the cloak structure. The magnetic probe (Fig. 1) was positioned 5 mm above the structure cloak. The probe was a homemade magnetic loop with diameter 5 mm and the loop was positioned perpendicular to the magnetic field of the emitter antenna. In this configuration, the magnetic probe measured a single component of the three Cartesian components of the magnetic field. In this case, this is the magnetic field perpendicular component to the loop that was measured. The magnetic loop scanned a 33 × 71 cm^2^ surface thanks to a 3D axis positioning system^36^.

The antenna and the probe were connected to a vector network analyzer^44^ that generate and measure the electromagnetic field. The electromagnetic field was emitted by the antenna and the probe only received the field.

The vector network analyzer measured the transmission coefficient between the emitter antenna and the magnetic probe. In our case, the transmission coefficient is equivalent to the magnetic field component perpendicular to the loop. The frequency range was from 1 to 6 GHz (our vector network works between 50 KHz and 6 GHz).

The complex transmission coefficient between the antenna and the probe is displayed in terms of magnitude in dB to visualize the intensity of the field. The real part of the magnetic field is also displayed since this is related to the phase of the field.

### Experimental set-up for acoustic waves

The acoustic wave experimental setup was positioned on an optical table with open space surrounded. A commercial loudspeaker [20 Hz–18 kHz] was placed at 20 cm from the structure cloak. The loud speaker was driven by a programmable functional generator (Tektronix AFG 3022B) and a power amplifier (Krohn-Hite Model 7500). A high sensitive microphone mounted on an x-y translation stage (Newmark NSL4) was used to record the two-dimensional acoustic pressure field distribution. The loudspeaker, acoustic cloak, and microphone were placed at the same level for characterizing how the pressure field was affected by the cloak. In the experiment, a five-period sinusoidal wave was launched by the loudspeaker. The time-dependent pre-amplified signal collected by the microphone was recorded by an oscilloscope (Agilent DSO 6104A) and downloaded to a computer for further analysis. The signal generation and acquisition were synchronized by the computer program. Therefore, we can map the 2D pressure field distribution with both amplitude and phase information. The scanning area is 25 × 25 cm^2^.

## Additional Information

**How to cite this article**: Xu, J. *et al*. Molding acoustic, electromagnetic and water waves with a single cloak. *Sci. Rep*. **5**, 10678; doi: 10.1038/srep10678 (2015).

## Supplementary Material

Supplementary Information

Supplementary Movie S1

Supplementary Movie S2

Supplementary Movie S3
