# Supplementary material for: Molding acoustic, electromagnetic and water waves with a single cloak
Source: Sci Rep. 2015 Jun 9;5:10678. doi: 10.1038/srep10678 (PMC4460817; doi:10.1038/srep10678)
Supplement: Supplementary Information [file srep10678-s1.pdf]

Molding acoustic, electromagnetic and water waves with a single cloak

Jun Xu, Xu Jiang, Nicholas Fang, Elodie Georget, Redha Abdeddaim, Jean-Michel Geffrin, Mohamed Farhat, Pierre Sabouroux, Stefan Enoch, Sébastien Guenneau

Supplementary information

Corresponding authors:
Prof. Nicholas Fang, Fax: 617-258-6156; E-mail: nicfang@mit.edu;
Dr. Sébastien Guenneau, Fax: +33 (0) 491-288-884; E-mail: sebastien.guenneau@fresnel.fr

S1. Numerical calculations

This supplemental material contains further numerical results performed with COMSOL Multiphysics (see Figures S1-S4) which exemplify that cloaking is achieved for different sizes and shapes of the obstacle (Figures S1&S2). This is in agreement with experiments done in MIT (which used a glass bottle of cross section as in Figure S1) and Institut Fresnel (where various metallic objects were tested, notably a square one as in Figure S2).

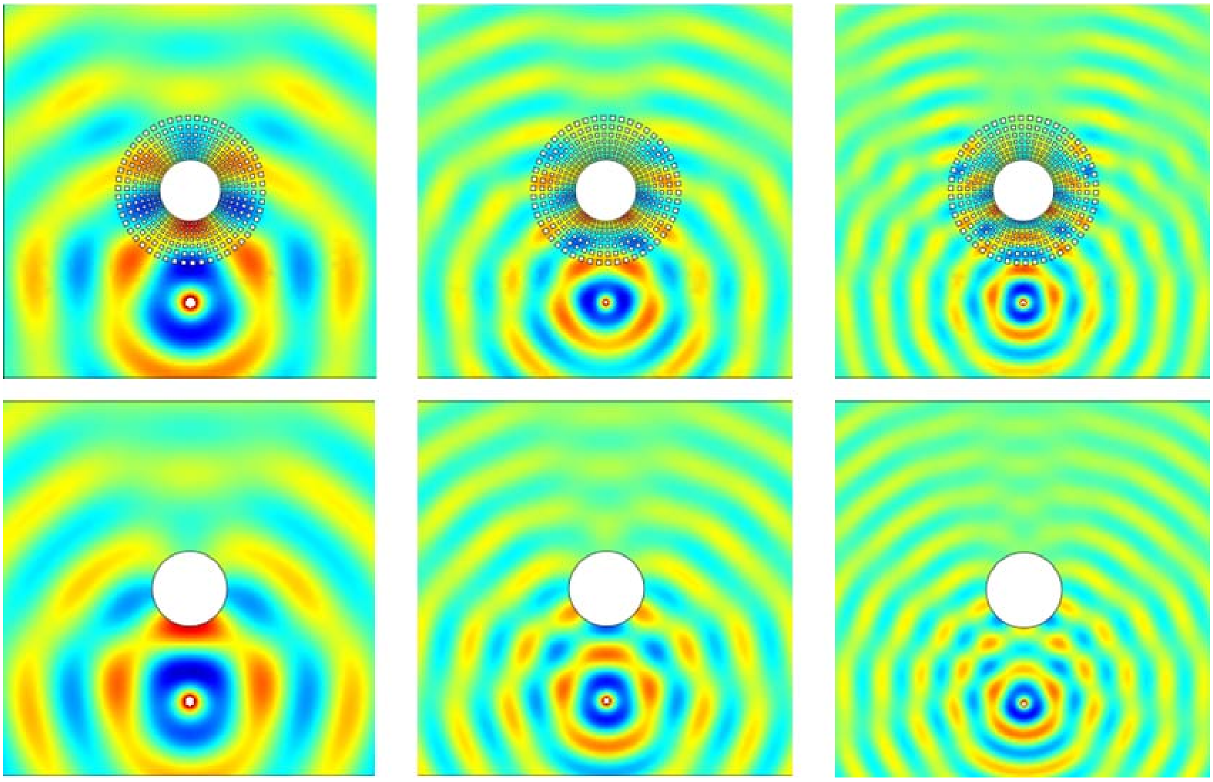

Fig. S1 Two-dimensional numerical results for acoustic (resp. microwave) cloaking: Plots of the real part of pressure field (resp. longitudinal magnetic field) for the glass bottle surrounded by the cloak (upper panels) and the glass bottle on its own (lower panels) for an acoustic source at 3 kHz (left column), 5 kHz (middle column) and 7 kHz (right column). The corresponding frequencies for transverse electric microwaves are 2.6 GHz, 4.3 GHz and 6.1 GHz.

The only constraint for cloaking is that the obstacle need be rigid in acoustics and metallic in microwaves (so as to meet the assumption of Neumann boundary conditions). One can see in Figures S2 that there is some field within the invisibility region, but it turns out that its amplitude is much reduced there in the experiments, see Figure 7 of main article.

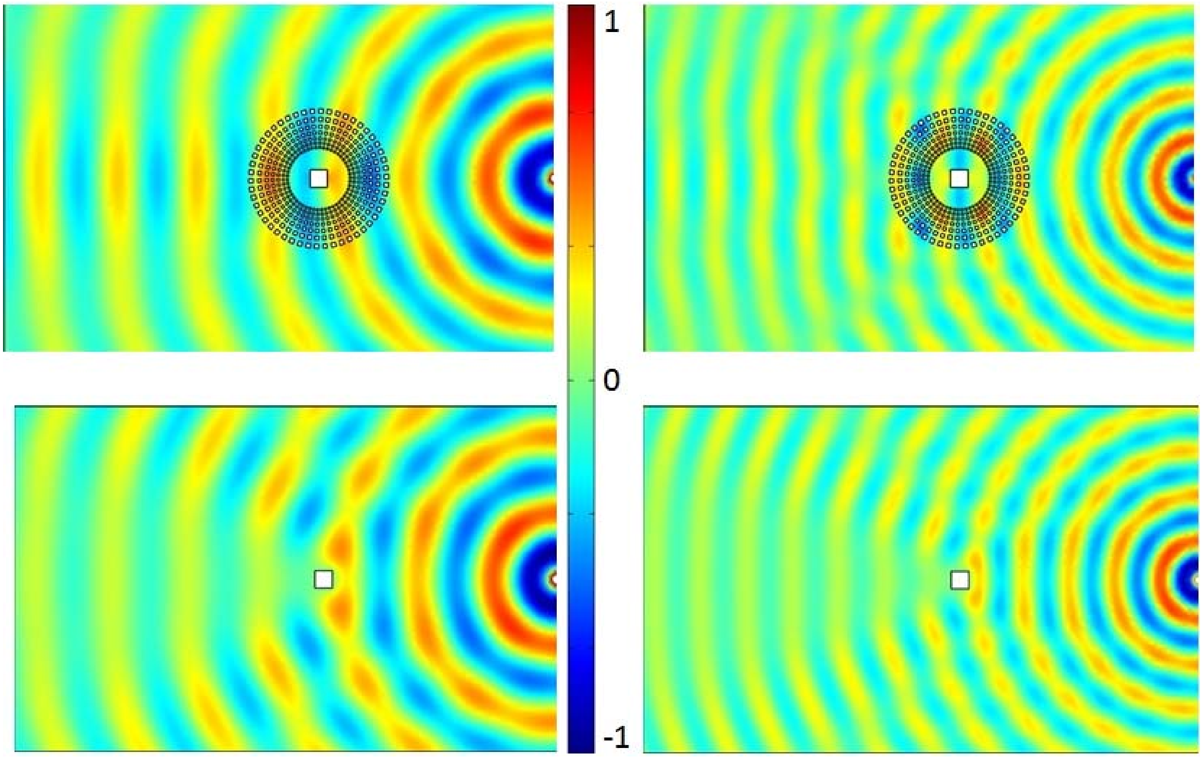

Fig. S2 **Two-dimensional numerical results for microwave (resp. acoustic) cloaking:** Plots of the real part of the longitudinal magnetic field **(resp. pressure field)** for a square metallic obstacle surrounded by the cloak (upper panels) and the square obstacle on its own (lower panels) for a ridged horn antenna generating a transverse electric wave at 3GHz (left column), 5.75GHz (right column). The corresponding frequencies for pressure waves are 3.45 kHz and 6.6 kHz. The reduced forward scattering (and fairly reduced backscattering) in upper panels can be attributed to the cloak. The plots in upper panels are also in good agreement with the lower panels in Fig. 9 of the main article.

Figures S3&S4 further confirm via computation of two-dimensional total radar cross section (RCS) the range of frequencies for cloaking of transverse electric and pressure acoustic waves (Figure S3) and surface water waves (Figure S4). It is also shown in these two figures that when the number of sectors constituting the cloak is larger, the RCS is further reduced, which is an hallmark of a homogenization process. One should nevertheless note that the lower bound of the frequency range for cloaking is not accurate in Figures S3&S4 since the 2D model breaks down when the wave wavelength becomes way larger than the cloak's height.

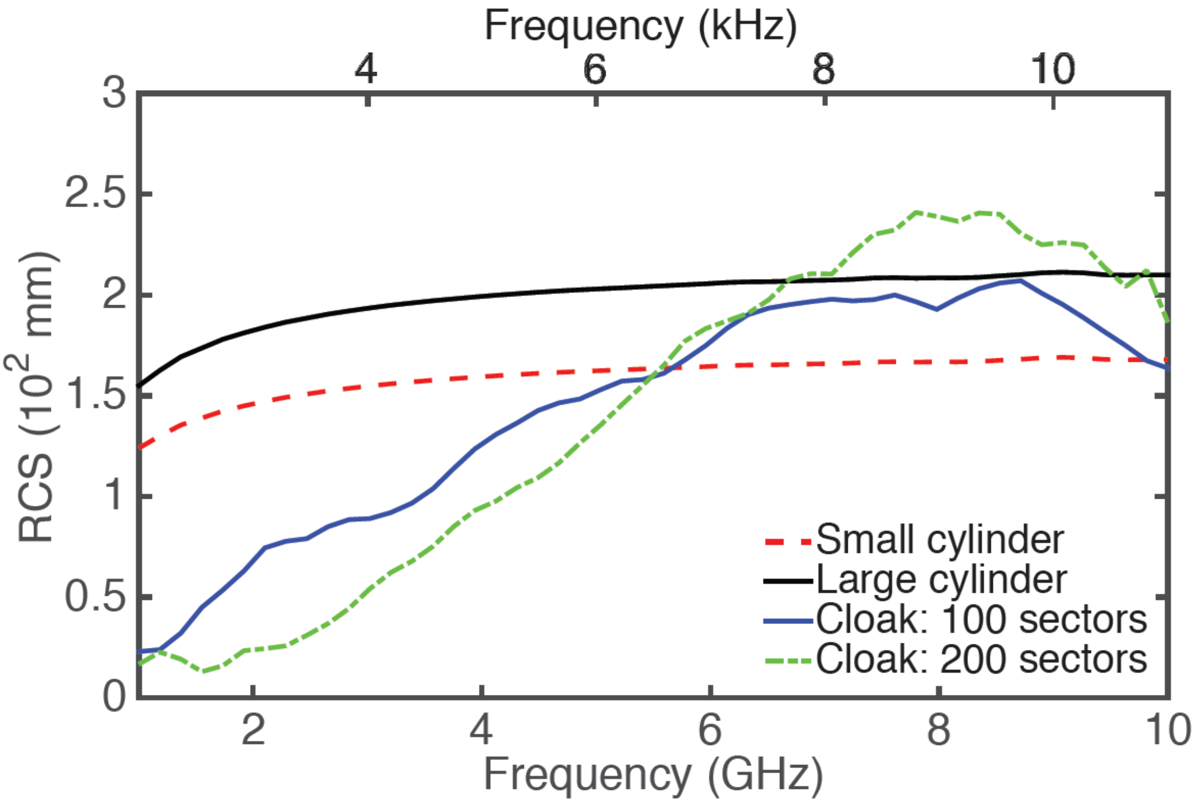

Fig. S3 Numerical results for total radar cross section of transverse magnetic and pressure waves: A small infinite conducting (resp. rigid) obstacle surrounded by cloak with 200 sectors (dashed green curve) is slightly below RCS of same obstacle surrounded by cloak with 100 sectors (solid blue curve), which is itself lower than RCS of small infinite conducting (resp. rigid) obstacle (dotted red curve), which is itself smaller than RCS of large infinite conducting (resp. rigid) obstacle of same diameter as the cloak (solid black curve) up to 7 GHz (resp. 8 kHz). One notes that the lower frequency bound for cloaking is not accurate since the wave wavelength becomes larger than the cloak's height (which is assumed to be invariant along the vertical direction in the 2D computations).

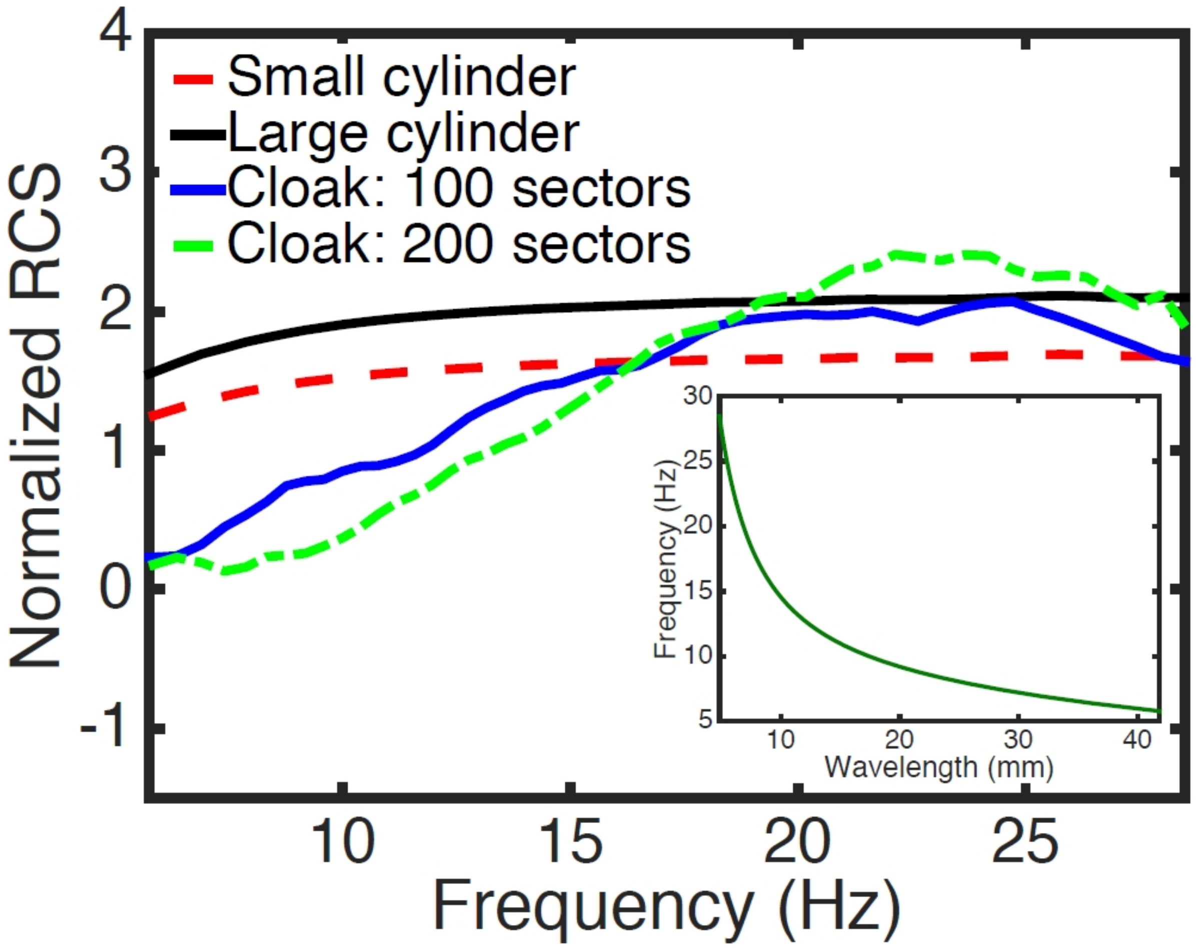

Fig. S4 Numerical results for total radar cross section of surface water waves: A small rigid (e.g. glass or metal) obstacle surrounded by cloak with 200 sectors (dashed green curve) is slightly below RCS of same obstacle surrounded by cloak with 100 sectors (solid blue curve), which is itself lower than RCS of small rigid obstacle (dotted red curve), which is itself smaller than RCS of large rigid obstacle of same diameter as the cloak (solid black curve) from 5 Hz to 16 Hz. An inset shows the dispersion of surface water waves in a liquid (methoxynonafluorobutane) with a depth of 9mm, with a capillarity 9.95mm, a small surface tension σ=13.6 N/cm and a large density ρ=1.529 g/mL.

S2. Acoustic experiments

In the experiment of the acoustic cloak, the 2D acoustic pressure field was measured by the 2D mapping system. The signal generation and acquisition were synchronized, so we can generate the time-resolved the information by assembling all the time-dependent signal collected at varied position. The movies S1, S2&S3 demonstrate how the acoustic pressure field, which was launched from the loudspeaker, propagates in the free space (movie S1) and is scattered by the obstacle (movie S2) and by the obstacle surrounded by the cloak (movie S3). The frequency of the acoustic wave in the measurement was 5 kHz with corresponding wavelength 16.9 cm. The loudspeaker was placed at 20 cm from the cloak. The pressure field distribution behind the cloak and the corresponding free space region were mapped. The area of the movie was 25*25 cm^2.

Movie description:

Movie S1 Acoustic experiment 1: Measured Real part of acoustic pressure wave propagating in free space at 5 kHz in the area marked with dashed lines shown in panel c of Fig. 4 of main article.

Movie S2 Acoustic experiment 2: Measured Real part of acoustic pressure wave scattered by the obstacle (glass bottle) at 5 kHz in the area marked with dashed lines shown in Fig. 4(c) of main article.

Movie S3 Acoustic experiment 3: Measured Real part of acoustic pressure wave scattered by the obstacle (a glass bottle) surrounded by the cloak at 5 kHz in the area marked with dashed lines shown in Fig. 4(c). The similarities between movies S1 and S3 are noted.
